# A systematic review of risk factors for neonatal mortality in Adolescent Mother’s in Sub Saharan Africa

**DOI:** 10.1186/1756-0500-7-750

**Published:** 2014-10-23

**Authors:** Astha Ramaiya, Ligia Kiss, Paula Baraitser, Godfrey Mbaruku, Zoe Hildon

**Affiliations:** Ifakara Health Institute, Dar-Es-Salaam, Tanzania; Department of Community Health and Prevention, Drexel University, 1121 N Barrett Lane, Newark, DE 19701 USA; London School of Hygiene and Tropical Medicine, London, UK; Kings College Hospital NHS Foundation Trust, London, UK; National University Singapore, Cheng San, Singapore

**Keywords:** Sub Saharan Africa, Neonatal mortality, Adolescent pregnancy, Adult pregnancy, Single, Risk factors

## Abstract

**Background:**

Worldwide, approximately 14 million mothers aged 15 – 19 years give birth annually. The number of teenage births in Sub Saharan Africa (SSA) is particularly high with an estimated 50% of mothers under the age of 20. Adolescent mothers have a significantly higher risk of neonatal mortality in comparison to adults. The objective of this review was to compare perinatal/neonatal mortality in Sub Saharan Africa and it’s associated risk factors between adolescents and adults.

**Results:**

We systematically searched six databases to determine risk factors for perinatal/neonatal mortality, and pregnancy outcomes, between adolescent and adults in SSA. Article’s quality was assessed and synthesized as a narrative. Being single and having a single parent household is more prevalent amongst adolescents than adults. Nearly *all* the adolescent mothers (97%) were raised in single parent households. These single life factors could be interconnected and catalyze other risky behaviors. Accordingly, having co-morbidities such as Sexually Transmitted Infections, or not going to school was more prevalent in younger mothers.

**Conclusions:**

Inter-generational support for single mothers in SSA communities appears essential in preventing both early pregnancies and ensuring healthy outcomes when they occur during adolescence. Future studies should test related hypothesis and seek to unpack the processes that underpin the relationships between being single and other risk indicators for neonatal mortality in young mothers. Current policy initiatives should account for the context of single African women’s lives, low opportunity, status and little access to supportive relationships, or practical help.

**Electronic supplementary material:**

The online version of this article (doi:10.1186/1756-0500-7-750) contains supplementary material, which is available to authorized users.

## Background

Forty one percent of all under five childhood deaths occur during the neonatal period (from birth to day 27). Reducing these deaths is one element of millennium development goal 4, which sets a target of reducing child mortality by two thirds [1–3]. Although, there has been an overall decline; this target is currently met by only 25% countries worldwide [4].

The top five causes of neonatal mortality worldwide are prematurity and low birth weight (LBW) (29%), neonatal infections (25%), birth asphyxia and birth trauma (23%), congenital anomalies (8%), neonatal tetanus (2%) and diarrhoeal disease (2%) [2].

Neonatal mortality is related to maternal age with young women having a higher risk of infant mortality (Additional file 1: Appendix 1). Worldwide, approximately 14 million adolescents aged 15 – 19 years give birth annually. Sixteen percent of young adults reside in SSA with a fertility rate of 112.84/1000 in comparison to 40.50/1000 in older women [5, 6]. Fifty percent of births in SSA are to mothers <20 years of age [5, 7]. Mothers <15, 16–17 years and 18–19 years have a 55%, 19% and 6% higher risk of neonatal mortality respectively in comparison to mothers ≥20 years [8].

Limited research has been conducted on the risk factors for neonatal mortality among young mothers in comparison with their older peers in SSA. A systematic review was conducted to determine the relationship between neonatal mortality and maternal age in SSA, focusing on indicators of socio-economic deprivation and lack of access to obstetric care.

## Methods

**Figure 1:**
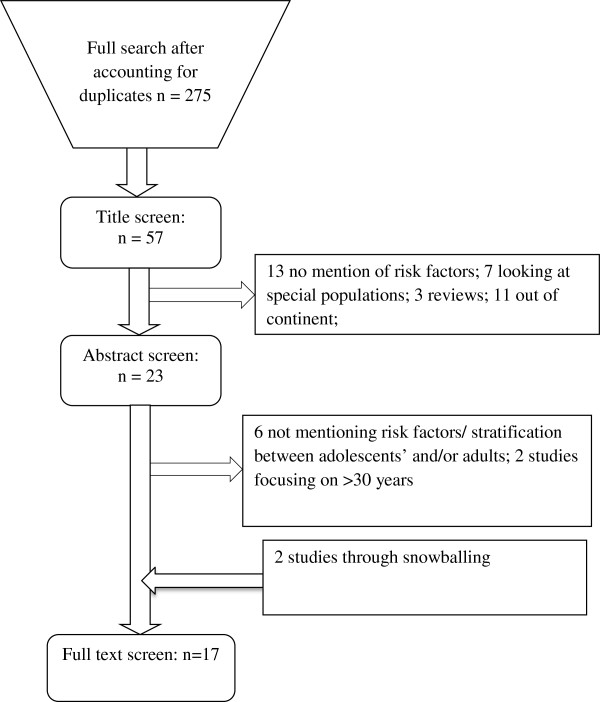
**Flow chart for literature screening & selection.**

Focusing on quantitative data measuring neonatal mortality between adolescents <20 years and mothers older than 20 years in SSA, we reviewed literature to determine risk of neonatal mortality stratified by maternal age. There was no existing prior review protocol.Studies were systematically screened from PubMed, Cochrane database, Adolec, Popline, Google Scholar and Global Health Archive on adolescent and adult pregnancy in SSA until February 2013 (Figure 1).

The studies were selected based on the main inclusion criteria: Any quantitative study measuring the association between risk factors for perinatal/neonatal mortality (PNM) and pregnancy outcome between adolescent and adults in SSA either comparing directly or as stratified groups. Due to the changing African developing context, we excluded publications prior to 1994. Also excluded were analyses that focused on older mothers, because neonatal mortality risk increases in mothers over 35 years of age, suggesting a potentially different set of risk indicators in this age category (Additional file 1: Appendix 3) [9–12].

Articles were graded using the Quality Assessment Tool for Quantitative Studies [13].

Randomized control trials and cohort studies were viewed as providing more robust findings, although cross-sectional studies were also examined. Risk factors for neonatal mortality were significant if p ≤ 0.05 and 95% Confidence Interval (95% CI) ≠ 0 were met. Articles were graded on the following scale: strong +++, moderate ++ and weak + by AR.

In order to calculate perinatal/neonatal mortality ratio (PNMR), data was extracted for adolescents and adults from individual studies and calculated using the formula: PNM cases/total number of adolescent or adult population*1000. A proportion of the rates was reported in the results.

Prevalence rate of risk factors was calculated through the extraction of data from individual studies, using the formula: total cases/total population of adolescent or adult population. If there were multiple studies reporting the same risk factor, the cases and population was totaled stratified by adolescent and adults. A proportion was determined for adolescents and adults in addition to the 95% CI and then a z score was calculated to determine the p-value. Some studies reported odds ratios (OR) and hazard ratios (HR); these were reported to provide additional statistics.

We report a narrative review comparing perinatal/neonatal mortality in SSA and it’s associated risk factors between mothers 15–19 years and 20–35 years of age.

## Results

A total of seventeen studies were included in the analysis (Additional file 1: Appendix 2). Forty one percent directly compared outcomes between adolescents and adults and another 59% stratified neonatal mortality or risk factor by age. Geographically, 35% were set in West Africa, 12% in Central Africa, 29% in East Africa, 12% in Southern Africa and 12% in SSA. Twenty nine percent of the studies were strong, 12% moderate and 47% rated weak (Table 1).

**Table 1:** **Comparison of risk factors for neonatal mortality between adolescents and adults**

Factors contribution to neonatal mortality	Measure	Adults (95%CI)	Adolescents (95%CI)	P-value	Reference & Study quality**
Perinatal/Neonatal Mortality	PNMR (/1000 live births)	23.47 (22.6 – 24.4)^	39.68 (37.3 – 42.1)^	≤0.01	14^+++^, 15^+++^,16^+^, 17^+^,18^+++^,19^+^,20^+++^,21^+^,22^+^,24^+++^,25^+++^
	Hazard Ratio	1	3.05 (1.3 – 6.81) 1.12 (0.92 – 1.36)		17^+^ 9^++^
	Odds Ratio	1	1.3 (0.3 – 6.4)		23^++^
Limited schooling (no/primary education)	Prevalence (%)	40.29 (35.45 – 45.13)^	75.93 (71.84 – 80.02)^	≤0.01	26^+++^, 28^++^
	Odds Ratio	1	4.9 (3.14 – 7.68)		28^++^
Single	Prevalence (%)	18.35 (14.52 – 22.16)^	47.65 (43.19 – 52.11)^	≤0.01	26^+++^,28^++^,27^+++^
	Odds Ratio	1	2.78 (1.65 – 4.70)		28^++^
≤2 - 3 ANC visits	Prevalence (%)	21.51 (19.35 – 23.67)^	22.16 (19.24 – 25.08)^	0.38	25^+++^, 26^+++^, 28^++^, 29^++^,
	OR	1.00	2.8 *(1.2 - 6.5)*		29^++^
Partner’s limited schooling (no/primary education)	Prevalence (%)	35.96 (30.08 – 41.92)	52.20 (45.68 – 58.72)	≤0.01	28^++^
	OR	1.00	1.95 (1.23 – 3.08)		28^++^
Parent’s not married	Prevalence (%)	38.54 (32.95 – 44.13)	97.34 (95.59 – 91.11)	≤0.01	28^++^
First pregnancy/primiparous	Prevalence	36.99 (27.7 – 46.28)^	79.82 (73.64 – 86.00)^	≤0.01	26^+++^,27^+++^,
Preterm delivery (<35-38 weeks)	Prevalence (%)	20.87 (19.14 – 22.60)^	44.04 (38.51 – 49.57)^	≤0.01	15^+++^, 17^+^, 29^++^
	OR	1.00	1.3 (0.6 – 2.6)		29^++^
Maternal co-morbidity*	Prevalence (%)	1.70 (1.0 – 2.42)^	7.60 (4.27 – 10.93)^	≤0.01	26^+++^, 27^+++^, 29^++^
	OR	1.00	5.9 (1.7 – 19.9)		
Pregnancy/labour complications^#^	Prevalence (%)	19.02 (13.95 – 24.05)^	29.82 (22.75 – 36.85)^	0.02	26^+++^, 27^+++^,
Very/ Low Birth Weight	Prevalence (%)	13.49 (12.26 – 14.72)^	23.05 (19.95 – 26.15)^	≤0.01	15^+++^, 17^+^, 25^+++^,27^+++^, 29^++^
	OR	1.00	2.9 *(1.5 - 5.6)*		29^++^

*Maternal co-morbidities: HIV, Malaria, Syphilis, TB, hyptertension, maternal infection.

#Pregnancy/labour complications: pre-eclamptic toxemia, eclampsia, premature rupture of membrane, ante-partum hemorrhage/post partum hemorrhage and obstructed labour.

**References listed following the order in which statistics appear, ratings are: +++strong; ++moderate, +weak;

^Average of studies.

Thirteen studies reported PNM across age categories [9, 14–25]. The PNMR between adolescents and adults was 39.68 deaths/1000 live births and 23.47 deaths/1000 live births respectively (p ≤ 0.01) [9, 14–25]. Three studies had OR (1.3 (95% CI: 0.3 – 6.4)) and HR (1.12 (95% CI: 0.92 – 1.36) & 3.05 (1.3 – 6.81)) reported [9, 17, 23]. Although all studies indicated a higher odds and prevalence of neonatal mortality amongst adolescents, there were two studies which showed non significant findings [9, 23].

Being single and having a single parent household (not living with partner/not married) was 2.6 and 2.5 times more prevalent amongst younger mothers than adults (p ≤ 0.01) respectively [26–28]. Strikingly, nearly *all* the adolescent mothers (97%) had grown up in single parent households. These single life factors appear to be key interrelated risk indicators that catalyze other risky behaviors. For instance, not going to school was 1.89 times more prevalent amongst younger mothers (p ≤ 0.01) [26, 28]; maternal comorbidity (HIV, syphilis, malaria, TB etc.) was a sizeable 4.5 times more likely in adolescents’, who may have multiple sexual partners, and risky lifestyles that expose them to transmittable diseases.

Although single status was quite likely for young mothers (47%), for those who were in relationships, partners education was also limited (52%) compared to older couples (36%, p ≤ 0.01) [28]. The socio-economic disadvantages of these circumstances appear to cascade into the birthing experience and physical outcomes of younger mother and their children.

Moreover, adolescents were 2.2 times more likely to give birth to their first child, compared to their adult counterparts (p ≤ 0.01) [26, 27]. It is therefore unsurprising that they are also 1.6 times more likely to face pregnancy and labour complications (pre-eclamptic toxemia, eclampsia, premature rupture of membrane, ante-partum hemorrhage/post partum hemorrhage and obstruction) (p ≤ 0.01) [26, 27, 29]. Prevalence of premature delivery between adolescents and adults was 44% and 21% (p ≤ 0.01) respectively [15, 17, 29], while the young mothers also had a 1.7 times greater chance of having a LBW neonate (p ≤ 0.01) [15, 17, 25, 27, 29].

## Discussion

Our review shows that younger mothers in SSA have increased social and biological risk factors associated with neonatal mortality. Although, analyses were limited by the heterogeneity of the study contexts, and some quality issues, this is the first analysis to pool studies and consider risk by maternal age. Moreover, given the diversity of included studies, we have found a remarkable consistency in data highlighting significant vulnerabilities of adolescent mothers.

Within SSA, the proportion of young women getting married has decreased, but the age of their first sexual encounter has stayed the same [30]. Reasons for this early sexual initiation include: lower socioeconomic status, lack of sexual education, friends sexual behavior, parental marital status and media/pornography access [31]. Within a developed country setting, father’s absence has shown to increase early sexual activity and consequently adolescent pregnancy [32], our study suggests that the same may be true in the developing context.

It has been demonstrated that in SSA (Ethiopia, Kenya, Malawi, Tanzania and Zimbabwe), it is not uncommon to become a single mother (total likelihood ranging from 30% - 68.8%) [33]. Moreover, the term ‘single mother family’ is viewed interchangeably with those households being particularly vulnerable [33]. For instance, the lack of family structure leads to worse child outcomes, intergenerational transmission of lower economic mobility, racial inequality and gender inequality [33, 34]. Our data appears to confirm that being a lone female parent will predispose daughters to follow the same circumstances, entrenching gendered vulnerabilities.

Nevertheless, our data did not cover all bases, and we would argue that overriding parental absence is the role of positive parental engagement, and appropriate handling of teenage pregnancies. Cultural understanding of this issue in SSA may lead to angry parenting, partners and community reactions. We know from qualitative studies, such as this one in Uganda [35], that teenage mothers may experiences a backlash to their circumstances, precisely because they are unmarried and dependent. As one teenage girl put it – “One may have a very harsh parent, myself I have a very harsh father. I fear that if I tell him he can beat me up. … in the community there are some aged women who recognize the pregnancy and talk about it” [35]. This study also mentioned that adolescents face difficulties in accessing and utilizing health services due to socioeconomic burden, stigmatization, lack of privacy and negative attitude from health staff.

Family support and return to school after childbearing is a protective factor towards good maternal and neonatal outcomes in teenagers [36, 37]. For example a study in South Africa has shown that if parent’s of the adolescent mother committed to taking care of the infant, adolescents would return to school and have a delay in the birth of the next child [37]. Yet, young mothers in SSA, as demonstrated in this report, are likely to have little partner support, or extended family to depend on, since they come from a single parent home.

We recommend that future studies should test related hypothesis and seek to unpack the processes that underpin the relationships between single status and other risk indicators for neonatal mortality in young SSA mothers. Current policy initiatives should consider the context of single African women’s lives, low opportunity, status and little access to supportive relationships, or practical help. For example, reinstating adolescent girls into school after the pregnancy increases health services utilization and reduces maternal co-morbidities, pregnancy complications and adverse neonatal outcomes.

## Conclusions

Overall, we have demonstrated that young mothers in these communities need to be recognized as having their own public health and intervention needs. Policy makers and health care providers need to account for family circumstances and make services more youth friendly and accessible. Crucially inter-generational education for single mothers in the community may stop the cycle of vulnerability of young women into early and/or risky pregnancies.

### Availability of supporting data

The data set supporting the results of this article is included within the article (and its additional file).

## Electronic supplementary material

Additional file 1: **Appendix 1: Factors influencing neonatal mortality within literature.** **Appendix 2.** Review of adolescent and adult risk factors and strength of evidence. **Appendix 3.** Search Strategy. (DOCX 96 KB)

## Abbreviations

95% CI: 95% Confidence interval
ANC: Antenatal care
HR: Hazard ratio
LBW: Low birth weight
OR: Odds ratio
PNM: Perinatal neonatal mortality
PNMR: Perinatal neonatal mortality rate
SSA: Sub Saharan Africa.
