# Supplementary material for: A systematic review of risk factors for neonatal mortality in Adolescent Mother’s in Sub Saharan Africa
Source: BMC Res Notes. 2014 Oct 23;7:750. doi: 10.1186/1756-0500-7-750 (PMC4216370; doi:10.1186/1756-0500-7-750)
Supplement: Supplementary file 1 — Additional file 1: Appendix 1: Factors influencing neonatal mortality within literature. Appendix 2. Review of adolescent and adult risk factors and strength of evidence. Appendix 3. Search Strategy. (DOCX 96 KB) [file 13104_2013_3272_MOESM1_ESM.docx]

*Appendix 1: Factors influencing neonatal mortality within literature*

| Maternal demographic characteristics | Education (1) |
| --- | --- |
|  | Maternal age: ≤ 15 – 20 years (2–9) and ≥30-40 years during delivery (10–12) |
|  | Polygamous household (13) |
|  | maternal height < 150 cm (14) |
|  | maternal socio economic status (1,10,15,16) |
|  | birth interval of less than 2 years (10) |
| Maternal behavior | late initiation of breast feeding after birth (17) |
|  | partial breastfeeding (17) |
|  | number of ANC visits during pregnancy (1,9,18) |
|  | maternal immunization (9) |
|  | multiple deliveries (13,19,20) |
| Maternal clinical outcome | maternal blood pressure (9,21) |
|  | HIV (22,23) |
|  | Parity (2,3,7,19,21,24) |
|  | Gravidity (25,26) |
|  | previous pregnancy outcome (14,21,26) |
|  | gestational weeks at birth (2,3) |
|  | maternal malaria at ANC (27) |
|  | maternal anemia [pact cell volume < 0.21] (2,26) |
|  | abnormal delivery [breech, other](2,20) |
|  | Obstructed labour (20,21,25,26) |
|  | rupture of membranes > 24 hours (21) |
|  | maternal temperature > 38^○^C (21) |
|  | pre-eclampsia/ eclampsia(24,26) |
|  | vaginal bleeding > 8 months (21) |
|  | maternal syphilis (14,28) |
| Neonatal clinical outcome | smaller and larger than averaged sized infants (10) |
|  | male infants (10) |
|  | low birth weight (2,3,7,18) |
|  | low Apgar score(4,7) |
| Environmental | birth during the dry season(19) |
| Health care provider | delivery by unskilled provider (9,29,30) |

*Appendix 2: Review of adolescent and adult risk factors and strength of evidence*

| **No** | **Lead author** | **Location** | **Sample size and description** | **Sample and study design** | **Analytic Methods** | **Risk factor for neonatal mortality amongst adolescents** | **Quality Score** | **Population** |
| --- | --- | --- | --- | --- | --- | --- | --- | --- |
|  | McCurdy, 2011 (9) | Sub Saharan Africa | 89,655, 15 – 49 year old women who had given birth within the last 3 years (Community and Facility based) | Retrospective Cohort Study | Neonatal Mortality Ratio | 12 – 19 v/s 20 - 29 had the highest neonatal mortality cases NMR: 30.9 v/s 17.4 | 1 | Stratification:  Maternal age |
|  | Diallo, 2010 (19) | Burkina Faso | 895 pregnant women followed up till 7 days after birth (Community based) | Prospective Cohort Study | Rate Ratio, X^2^ , Logistic Regression | Perinatal Mortality Ratio:  < 20 years: 147.7/1000  20 – 35 year: 66.8/1000 | 1 | Stratification:  Maternal age |
|  | Kurth, 2010 (18) | Gabon | 775 women delivering in 3 health facilities with women aged 13 – 45 years (Facility based) | Cross Sectional | Univariable analysis, Multivarabile logistic regression analysis | a. Adolescent:   1. Increased risk to deliver a baby with low birth weight (OR: 2.9; 95% CI: 1.5–5.6). 2. Less likely to attend antenatal care visits (< 3 consultations) in comparison to adults (2.8 (1.2 – 6.5) ). | 2 | Adolesc v/s adult :  LBW  ANC visit |
|  | Wasunna, 2002 (1) | Kenya | 142 mothers and LBW newborn neonates dyads were recruited (Facility Based) | Cross sectional descriptive study between mothers < 20 years and > 20 years | Χ^2^ | Adolescents more likely to:  a. have less formal education (36.6% v/s 71.1%) (p<0.01)  b. to be single/unmarried (75.4% v/s 32.9%) (p<0.01)  c. primigravidae (76.8% v/s 37%) (p<0.01)  d. Inadequate (< 50% of attendance required at birth) ANC attendance (73.9% v/s 47.9%) (p=0.04) | 3 | Adolesc v/s adults : LBW newborns :  Education  Single  Primigravid  ANC attendance |
|  | Wasunna, 2002 (3) | Kenya | 142 neonates < 2000 grams were studied and followed upto death, discharge from hospital or upto 1 month at the hospital (Facility Based) | Cross sectional descriptive study between mothers < 20 years and > 20 years | Graphs, Risk Ratio, p value | Adolescent mothers were more likely:  a. to deliver within the 30^th^ week because of delivering LBW neonates (47.8% v/s 26.0%) (p=0.02)  b. to have LBW (49.2% v/s 30%) (p=0.01)  c. Respiratory distress (RR: 1.25 (1.04 – 1.50)  d. Increased mortality (57.7% v/s 42.3%) (p=0.04) | 3 | Adolesc v/s adult:  Deliver earlier  LBW  Respir distress  Mortality |
|  | Nkwabong, 2009 (4) | Cameroon | 193 teenagers and 403 adults between 20 – 25 years with a total of 2315 deliveries (Facility Based) | Retrospective Chart Review | t-test and Fisher’s exact test | Adolescents had:   - 1. Increased neonatal mortality within the age group of <= 15 years OR 29.6 (27.9 – 31.2) | 3 | Adolesc v/s Adult :  Early neonatal death |
|  | Adeyinka, 2010 (24) | Nigeria | 45 adolescents < 18 years and 90 adults between 20 – 35 years (Facility based) | Case Control Study | X^2^, T-Test | Adolesc v/s Adult:   - 1. Any complication (p<0.01)   2. Eclampsia (20% v/s 3% p<0.01)   Adolescent only:   - 1. 95% single   2. 84% primiparous | 3 | Adolesc v/s adult :  Any complication  Eclampsia |
|  | Ekure, 2011 (7) | Nigeria | 603 babies weighing ≥500 grams and 20 weeks pregnant (Facility Based) | Prospective Audit | Descriptive & multivariable regression | Neonatal mortality:  15 – 19 years: 3/7 (42.9%)  20 – 29 years: 20/228 (8.8%) | 3 | Stratification:  Maternal age |
|  | Kim, 2012 (6) | Zambia | 1229 HIV positive women | Randomized Clinical Trial | OR | Maternal age < 20 (HR: 3.05 (1.3 – 6.81) | 1 | Stratification:  Maternal age < 20 |
|  | Weiner, 2003 (8) | Kenya | 910 births (Facility Based) | Cross sectional | OR | Neonatal mortality rate:  < 20 years: 124.3/1000 | 3 | Stratification:  NMR maternal age < 20 : 124.3/1000 |
|  | Engmann, 2012 (31) | Ghana | 24,497 pregnant women (Community based). | Prospective Cohort Study | Perinatal Mortality Ratio | PNMR: <20 years : 56.2/1000 live births, 20-34: 34.3/1000 | 1 | Stratification: NMR maternal age < 20 v/s adults |
|  | Taffa, 2003 (32) | Kenya | 226 adolescents <20 years,205 adults 20 – 35 years (Community based) | Comparative Study cross sectional survey | OR | No education/primary school (AOR: 4.9 (3.14 – 7.68)) Adolescents: 180/226, Adults: 91/205.  Never been married (AOR 2.78 (1.65, 4.70))  Adolescents: 67/226, Adults: 27/205  Parents educational level (AOR 1.95 (1.23 – 3.08))  Adolescents: 83/226, Adults: 64/205  Parents not married (AOR 0.04 (0.02-0.11))  Adolescents: 220/226, Adults: 79/205 | 2 | Adolescent v/s adult :  Significant:  Education  Never married  Parents no education/primary  Parents not married  Non significant:  ANC attended (216/226 v/s 197/205) |
|  | Diallo, 2011 (13) | Burkina Faso | 1162 pregnant women identified (community based) | Prospective cohort study | OR | No risk factors | 1 | Stratification NM: adolescents <20 years v/s adults 20-35 (AOR 1.3 (0.6 – 3.1) v/s 1.00)  <20 neonatal mortality: 8/133  20 – 25 neonatal mortality: 28/642 |
|  | Kulmala, 2011 (2) | Malawi | 795 pregnant women and 813 fetuses (facility based) | Prospective cohort study | OR | No risk factors | 2 | Stratification NM: adolescents < 20 years v/s adults 20 – 30 years (AOR: 1.3 (0.3 – 6.4)) |
| 1. T | Titaely, 2000 (10) | Sub Saharan Africa | 185,383 women and 100,683 live singleton births (community based) | Retrospective cohort study using Demographic Health Survey | HR | No risk factors | 2 | Stratification NM: adolescent <20 years: 1.12 (0.92 – 1.36), 20 – 29: 1.00 |
|  | Onayade, 2006 (33) | Nigeria | 470 records, 235 neonatal deaths and 235 admitted neonates (facility based) | Comparative retrospective chart review | Descriptive & prevalence | No risk factor | 3 | Stratification NMR:  Adolescent <20 years: 33/55 (600/1000 live births), 20-29: 189/356 (530/1000 live births) |
|  | Van Dillen, 2008 (34) | Namibia | 598 singleton births from primiparous women (facility based) | Comparative retrospective chart review | Descriptive & prevalence | No risk factor | 3 | Adolescent v/s adult:  LBW: 24/227 v/s 51/371  Neonatal death: 2/227 v/s 4/371  < 3 ANC visits:  46/227 v/s 57/371 |

*Appendix 3: Search Strategy*

| **Database** | **Keywords** | **Total articles** | **Title** | **Abstract** | **Full text** |
| --- | --- | --- | --- | --- | --- |
| Pubmed | "neonatal mortality" AND " Sub Saharan Africa" | 36 | 14 (3 not in English, 19 didn’t have neonatal/infant/perinatal mortality or adverse pregnancy in the title) | 8 (5 no risk factor, 1 looking at causes of mortality in twins) | 5(McCurdy, 2011; Diallo, 2010; Kurth 2010; Kulmala, 2000, Titaley, 2010)( 3 no stratification by maternal age) |
|  | "Africa South of the Sahara/epidemiology"[Mesh]AND "maternal health" AND "neonatal health" AND "health systems" | 1 | 1 | 1 | 0 (1 no stratification by maternal age) |
|  | (“Teenage pregnancy” AND “neonatal mortality”) | 8 | 5 ( 1 review, 2 not having neonatal/perinatal mortality/outcome) | 1 (4 not in SSA) | 1 (Onayade,2006) |
|  | ("Pregnancy in Adolescence"[Mesh] AND "Africa South of the Sahara"[Mesh]) AND "Infant Mortality"[Mesh]) | 8 | 7 | 5(1 different population, 1 no risk factor) | 5(Nkwabong, 2009; wasunna, 2002;wasunna,2002, Taffa, 2003, VanDillen, 2008 ) |
|  | "neonatal mortality" AND "maternal HIV" AND "Africa South of the Sahara/epidemiology"[Mesh] | 3 | 1 (1 different population, 1 repeat) | 0(1 different population) | 0 |
|  | "neonatal mortality" AND "maternal HIV" | 6 | 2 (4 repeat) | 1 (1 review) | 1 (Kim, 2012) |
|  | ((("Pregnancy in Adolescence"[Mesh] AND "Risk Factors"[Mesh]) AND "Infant Mortality"[Mesh])) AND "Africa South of the Sahara"[Mesh] | 3 | (3 repeat) |  |  |
|  | ("pregnancy in adolescence"[mesh] and "africa south of the sahara"[mesh]) and "infant mortality"[mesh] AND "low birth weight | 4 | (3 repeat, 1 no mention of mortality) | 0 | 0 |
|  | "pregnancy in adolescence"[mesh] and "africa south of the sahara"[mesh]) and "infant mortality"[mesh] AND "parity" | 1 | (1 repeat) |  |  |
|  | "pregnancy in adolescence"[mesh] and "africa south of the sahara"[mesh]) and "infant mortality"[mesh] AND ("gestational age" OR “preterm”) | 2 | (2 repeat) |  |  |
|  | "pregnancy in adolescence"[mesh] and "infant mortality"[mesh] AND “gravidity” | 2 | 2 | 0(2 out of continent) | 0 |
|  | Pregnancy in Adolescence"[Mesh]) AND "neonatal mortality" AND "risk factors" AND "africa south of the sahara"[mesh]) | 3 | 1 (2 repeat) | 1 | 1 (Adeyinka, 2010) |
|  | ("maternal malaria" AND "neonatal mortality") AND "Africa South of the Sahara"[Mesh] | 4 | 2(1 repeat, 1 no mention of mortality or neonatal outcome) | 1 (1 looking at neonatal malaria status) | 0 (1 no stratification by maternal age) |
|  | (("Infant Mortality"[Mesh]) AND "Malaria"[Mesh]) AND "Africa South of the Sahara/epidemiology"[Mesh] | 45 | 9 (2 repeat, 33 no mention of neonatal mortality or outcome) | 1(4 not focusing on neonatal mortality, 1 no risk factor, 2 review, 1 looking at various risk factors together, | 1 ( Weiner, 2003) |
|  | "maternal smoking" AND "neonatal mortality" AND "Africa South of the Sahara/epidemiology"[Mesh] | 0 | 0 | 0 | 0 |
|  | "maternal smoking" AND "neonatal mortality" | 16 | 2 (14 not mentioning neonatal/perinatal mortality) | (2 out of continent) | 0 |
|  | "anemia" AND "neonatal mortality" AND "africa south of sahara" | 4 | 2 (1infant mortality, 1 no mention of mortality) | 0 (1 dealing with beneficial factor, 1 no mention of adolescent risk factors) | 0 |
|  | "maternal weight" AND "neonatal mortality" AND "Africa" | 0 | 0 | 0 | 0 |
|  | (("Infant Mortality"[Mesh] AND "Maternal Age"[Mesh])) AND "Africa South of the Sahara"[Mesh] | 55 | 9 (5 repeat, 41 not mentioning neonatal/perinatal mortality) | 4 (1 looking at perinatal mortality; 3 not in SSA; 1 no risk factors; | 1 (Ekure, 2011) (1 no stratification by maternal age; 2 studies amongst women > 30 years) |
|  | "Maternal Age"[Mesh] AND "Infant Mortality"[Mesh] AND "Parity"[Mesh] AND "Africa South of the Sahara"[Mesh]) | 16 | 0(7 repeat, 9 not mentioning neonatal/perinatal mortality) |  |  |
| Snowballing |  |  |  |  | 2 (Engmann, 2012; Diallo, 2011) |

References:

1. Wasunna A, Mohammed K. Low birthweight babies: socio-demographic and obstetric characteristics of adolescent mothers at Kenyatta National Hospital, Nairobi. East Afr Med J [Internet]. 2002 Oct [cited 2012 Feb 23];79(10):543–6. Available from: http://www.ncbi.nlm.nih.gov/pubmed/12635761

2. Kulmala T, Vaahtera M, Ndekha M, Koivisto AM, Cullinan T, Salin ML, et al. The importance of preterm births for peri- and neonatal mortality in rural Malawi. Paediatr Perinat Epidemiol [Internet]. 2000 Jul [cited 2012 Feb 22];14(3):219–26. Available from: http://www.ncbi.nlm.nih.gov/pubmed/10949213

3. Wasunna A, Mohammed K. Morbidity and outcome of low birthweight babies of adolescent mothers at Kenyatta National Hospital, Nairobi. East Afr Med J. 2002;79(10):539–42.

4. Nkwabong E, Fomulu JN. Adolescent Pregnancies and Deliveries: Problems Encountered. Trop Doct [Internet]. Gynaecology & Obstetric Service, University Teaching Hospital Yaoundé, Yaoundé, Cameroon enkwabong@yahoo.fr DOI - 10.1258/td.2008.080047 SRC - Pubmed ID2 - 19211412 FG - 0; 2009;39(1):9–11. Available from: http://www.ncbi.nlm.nih.gov/pubmed/19211412

5. Iloki L-H, Koubaka R, Itoua C, Mbemba Moutounou G-M. [Teenage pregnancy and delivery: 276 cases observed at the Brazzaville University Hospital, Congo]. J Gynecol Obstet Biol Reprod (Paris) [Internet]. 2004 Feb [cited 2012 Feb 23];33(1 Pt 1):37–42. Available from: http://www.ncbi.nlm.nih.gov/pubmed/14968053

6. Kim H-Y, Kasonde P, Mwiya M, Thea DM, Kankasa C, Sinkala M, et al. Pregnancy loss and role of infant HIV status on perinatal mortality among HIV-infected women. BMC Pediatr [Internet]. 2012 Aug 31 [cited 2012 Oct 2];12(1):138. Available from: http://www.ncbi.nlm.nih.gov/pubmed/22937874

7. Ekure EN, Ezeaka VC, Iroha E, Egri-Okwaji M. Prospective audit of perinatal mortality among inborn babies in a tertiary health center in Lagos, Nigeria. Niger J Clin Pract [Internet]. 2011 [cited 2012 Jul 12];14(1):88–94. Available from: http://www.ncbi.nlm.nih.gov/pubmed/21494000

8. Weiner R, Ronsmans C, Dorman E, Jilo H, Muhoro A, Shulman C. Labour complications remain the most important risk factors for perinatal mortality in rural Kenya. Bull World Health Organ [Internet]. 2003 Jan [cited 2012 Oct 3];81(8):561–6. Available from: http://www.scielosp.org/pdf/bwho/v81n8/v81n8a05.pdf

9. McCurdy RJ, Kjerulff KH, Zhu J. Prenatal care associated with reduction of neonatal mortality in Sub-Saharan Africa: evidence from Demographic and Health Surveys. Acta Obstet Gynecol Scand [Internet]. 2011 Jul [cited 2012 Feb 11];90(7):779–90. Available from: http://www.ncbi.nlm.nih.gov/pubmed/21426311

10. Titaley CR, Dibley MJ, Roberts CL, Agho K. Combined iron/folic acid supplements and malaria prophylaxis reduce neonatal mortality in 19 sub-Saharan African countries. Am J Clin Nutr [Internet]. 2010 Jul [cited 2012 Feb 22];92(1):235–43. Available from: http://www.ncbi.nlm.nih.gov/pubmed/20504976

11. Oboro VO, Dare FO. Pregnancy Outcome in Nulliparous Women Aged 35 or Older. West Afr J Med [Internet]. Department of Obstetrics and Gynaecology, LAUTECH College of Health Sciences, PMB 4400, Osogbo, Osun State, Nigeria. oborovo@yahoo.com SRC - Pubmed ID2 - 16722362 FG - 0; 2006;25(1):65–8. Available from: http://www.ajol.info/index.php/wajm/article/viewFile/28248/5016

12. Marai W, Lakew Z. Pregnancy Outcome in the Elderly Gravida in Addis Ababa. East Afr Med J [Internet]. Department of Obstetrics and Gynaecology, Jimma University, Addis Ababa, Ethiopia. SRC - Pubmed ID2 - 12380868 FG - 0; 2002;79(1):34–7. Available from: http://www.ajol.info/index.php/eamj/article/viewFile/8922/1749

13. Diallo A, Meda N, Ouedraogo W, Cousens S, Tylleskar T. A prospective study on neonatal mortality and its predictors in a rural area in Burkina Faso: can MDG-4 be met by 2015? J Perinatol. 2011 Oct;31(10):656–63.

14. McDermott J, Steketee R, Wirima J. Perinatal mortality in rural Malawi. Bull World Health Organ [Internet]. 1996 Jan [cited 2012 Jul 13];74(2):165–71. Available from: http://www.pubmedcentral.nih.gov/articlerender.fcgi?artid=2486898&tool=pmcentrez&rendertype=abstract

15. Taffa N, Omollo D, Matthews Z. Teenage Pregnancy Experiences in Rural Kenya. Int J Adolesc Med Health [Internet]. African Population and Health Research Center (APHRC), Shelter Afrique Center, P. O. Box 10787, 00100 GPO, Nairobi, Kenya. ntaffa@aphrc.org SRC - Pubmed ID2 - 14719415 FG - 0; 2003;15(4):331–40. Available from: http://www.ncbi.nlm.nih.gov/pubmed?term=Teenage pregnancy experiences in rural Kenya.

16. Fenn B, Kirkwood BR, Popatia Z, Bradley DJ. Inequities in neonatal survival interventions: evidence from national surveys. Arch Dis Child Fetal Neonatal Ed [Internet]. 2007 Sep [cited 2012 Mar 1];92(5):F361–6. Available from: http://www.pubmedcentral.nih.gov/articlerender.fcgi?artid=2675357&tool=pmcentrez&rendertype=abstract

17. Edmond KM, Zandoh C, Quigley MA, Amenga-Etego S, Owusu-Agyei S, Kirkwood BR. Delayed breastfeeding initiation increases risk of neonatal mortality. Pediatrics [Internet]. 2006 Mar [cited 2011 Aug 24];117(3):e380–6. Available from: http://www.ncbi.nlm.nih.gov/pubmed/16510618

18. Kurth F, Bélard S, Mombo-Ngoma G, Schuster K, Adegnika AA, Bouyou-Akotet MK, et al. Adolescence as Risk Factor for Adverse Pregnancy Outcome in Central Africa--A Cross-Sectional Study. PLoS One [Internet]. Medical Research Unit, Albert Schweitzer Hospital, Lambaréné, Gabon. DOI - 10.1371/journal.pone.0014367 SRC - Pubmed ID2 - 21188301 FG - 0; 2010;5(12):e14367. Available from: http://www.ncbi.nlm.nih.gov/pubmed/21188301

19. Diallo AH, Meda N, Zabsonré E, Sommerfelt H, Cousens S, Tylleskär T. Perinatal mortality in rural Burkina Faso: a prospective community-based cohort study. BMC Pregnancy Childbirth [Internet]. 2010 Jan [cited 2012 Feb 22];10:45. Available from: http://www.pubmedcentral.nih.gov/articlerender.fcgi?artid=2931454&tool=pmcentrez&rendertype=abstract

20. Vanneste AM, Ronsmans C, Chakraborty J, De Francisco A. Prenatal screening in rural Bangladesh: from prediction to care. Health Policy Plan [Internet]. 2000 Mar [cited 2012 Jul 13];15(1):1–10. Available from: http://www.ncbi.nlm.nih.gov/pubmed/10731229

21. Chalumeau M, Salanave B, Bouvier-Colle MH, de Bernis L, Prual A, Bréart G. Risk factors for perinatal mortality in West Africa: a population-based study of 20326 pregnancies. MOMA group. Acta Paediatr [Internet]. 2000 Sep [cited 2012 Jul 12];89(9):1115–21. Available from: http://www.ncbi.nlm.nih.gov/pubmed/11071095

22. Gichuhi C, Obimbo E, Mbori-Ngacha D, Mwatha A, Otieno P, Farquhar C, et al. Predictors of Mortality in HIV-1 Exposed Uninfected Post-Neonatal Infants at the Kenyatta National Hospital, Nairobi. East Afr Med J [Internet]. Department of Clinical Pharmacology and Therapeutics, College of Health Sciences, University of Nairobi, P.O. Box 19676, Nairobi, Kenya. SRC - Pubmed ID2 - 16619717 FG - 0; 2005;82(9):447–51. Available from: http://www.ncbi.nlm.nih.gov/pubmed?term=Predictors of mortality in HIV-1 exposed uninfected post-neonatal infants at the Kenyatta National Hospital, Nairobi.

23. Brocklehurst P, French R. The Association Between Maternal HIV Infection and Perinatal Outcome: A Systematic Review of the Literature and Meta-Analysis. Br J Obstet Gynaecol [Internet]. National Perinatal Epidemiology Unit, Radcliffe Infirmary, Oxford, UK. SRC - Pubmed ID2 - 9746375 FG - 0; 1998;105(8):836–48. Available from: http://www.ncbi.nlm.nih.gov/pubmed?term=The association between maternal HIV infection and perinatal outcome: a systematic review of the literature and meta-analysis

24. Adeyinka DA, Oladimeji O, Adekanbi TI, Adeyinka FE, Falope Y, Aimakhu C. Outcome of adolescent pregnancies in southwestern Nigeria: a case-control study. J Matern Fetal Neonatal Med [Internet]. 2010 Aug [cited 2011 Dec 31];23(8):785–9. Available from: http://www.ncbi.nlm.nih.gov/pubmed/20082596

25. Vaahtera M, Kulmala T, Ndekha M, Koivisto AM, Cullinan T, Salin ML, et al. Antenatal and Perinatal Predictors of Infant Mortality in Rural Malawi. Arch Dis Child Fetal Neonatal Ed [Internet]. Medical School, University of Tampere, PO Box 607, FIN-33101 Tampere, Finland. merimaaria.vaahtera@kolumbus.fi SRC - Pubmed ID2 - 10794786 FG - 0; 2000;82(3):F200–4. Available from: http://www.ncbi.nlm.nih.gov/pubmed?term=Antenatal and perinatal predictors of infant mortality in rural Malawi.

26. Kulmala T, Vaahtera M, Rannikko J, Ndekha M, Cullinan T, Salin ML, et al. The relationship between antenatal risk characteristics, place of delivery and adverse delivery outcome in rural Malawi. Acta Obstet Gynecol Scand [Internet]. 2000 Nov [cited 2012 Jul 13];79(11):984–90. Available from: http://www.ncbi.nlm.nih.gov/pubmed/11081685

27. Menéndez C, Bardají A, Sigauque B, Sanz S, Aponte JJ, Mabunda S, et al. Malaria prevention with IPTp during pregnancy reduces neonatal mortality. PLoS One [Internet]. 2010 Jan [cited 2011 Aug 30];5(2):e9438. Available from: http://www.pubmedcentral.nih.gov/articlerender.fcgi?artid=2829080&tool=pmcentrez&rendertype=abstract

28. Bloland P, Slutsker L, Steketee RW, Wirima JJ, Heymann DL, Breman JG. Rates and risk factors for mortality during the first two years of life in rural Malawi. Am J Trop Med Hyg [Internet]. 1996 Jan [cited 2012 Feb 23];55(1 Suppl):82–6. Available from: http://www.ncbi.nlm.nih.gov/pubmed/8702044

29. Penfold S, Hill Z, Mrisho M, Manzi F, Tanner M, Mshinda H, et al. A Large Cross-Sectional Community-Based Study of Newborn Care Practices in Southern Tanzania. PLoS One [Internet]. Faculty of Infectious and Tropical Diseases, London School of Hygiene and Tropical Medicine, London, United Kingdom. Suzanne.penfold@lshtm.ac.uk DOI - 10.1371/journal.pone.0015593 SRC - Pubmed ID2 - 21203574 FG - 0; 2010;5(12):e15593. Available from: http://www.ncbi.nlm.nih.gov/pubmed?term=A large cross-sectional community-based study of newborn care practices in southern Tanzania.

30. Adam T, Lim SS, Mehta S, Bhutta ZA, Fogstad H, Mathai M, et al. Cost effectiveness analysis of strategies for maternal and neonatal health in developing countries. BMJ [Internet]. 2005 Nov 12 [cited 2011 Aug 7];331(7525):1107. Available from: http://www.pubmedcentral.nih.gov/articlerender.fcgi?artid=1283271&tool=pmcentrez&rendertype=abstract

31. Engmann C, Walega P, Aborigo R a, Adongo P, Moyer C a, Lavasani L, et al. Stillbirths and early neonatal mortality in rural Northern Ghana. Trop Med Int Health [Internet]. 2012 Mar [cited 2013 May 23];17(3):272–82. Available from: http://www.ncbi.nlm.nih.gov/pubmed/22175764

32. Taffa N. A comparison of pregnancy and child health outcomes between teenage and adult mothers in the slums of Nairobi, Kenya. Int J Adolesc Med Health [Internet]. 2003;15(4):321–9. Available from: http://www.ncbi.nlm.nih.gov/pubmed/14719414

33. Onayade AA, Sule SS, Elusiyan JBC. Determinants of neonatal mortality at Wesley Guild Hospital, Ilesa, Nigeria. Niger J Med [Internet]. 2006 [cited 2013 Feb 22];15(3):271–6. Available from: http://www.ncbi.nlm.nih.gov/pubmed/17111757

34. Van Dillen J, van Beijeren E, van Roosmalen J. Perinatal Outcome of Primiparous Teenagers in Northern Namibia. Trop Doct [Internet]. Department of Obstetrics, Leiden University Medical Centre, Leiden, Postbus 9600, 2300 RC Leiden, The Netherlands. gwamupanda@caiway.nl DOI - 10.1258/td.2007.070093 SRC - Pubmed ID2 - 18453514 FG - 0; 2008;38(2):122–5. Available from: http://www.ncbi.nlm.nih.gov/pubmed?term=Perinatal outcome of primiparous teenagers in northern Namibia.
